# Biochemical Content of Cambium of* Abies nephrolepis* Eaten by Bears on the Far East of Russia

**DOI:** 10.1155/2017/3020571

**Published:** 2017-04-26

**Authors:** I. V. Seryodkin, A. M. Zakharenko, P. S. Dmitrenok, K. S. Golokhvast

**Affiliations:** ^1^Pacific Geographical Institute FEB RAS, 7 Radio St., Vladivostok 690041, Russia; ^2^Far Eastern Federal University, 8 Sukhanova St., Vladivostok 690091, Russia; ^3^G.B. Elyakov Pacific Institute of Bioorganic Chemistry, 159 Prospect 100 Let Vladivostoku, Vladivostok 690022, Russia

## Abstract

The peculiarity of bears behavior of stripping of bark is typical for all species. We have described the damage to trees, by Asiatic black bear* (Ursus thibetanus)* and brown bear* (U. arctos)* in Primorsky Krai and by brown bears on the Sakhalin Island during 1998–2015. In this study, we studied the damaged bark of the tree only in cases where it was clear that part of the cambium was eaten by bears. Cambium of species* Abies nephrolepis* is the most preferred for bear consumption in Primorsky Krai. We distinguished very large seasonal fluctuations in the amount of its consumption. The greatest interest of bears in this kind of food is in the summer time. We have analyzed the composition of the cambium of* A. nephrolepis*. These results suggest that the important purpose of the use of this kind of food is to restore and maintain the normal functioning of the intestines.

## 1. Introduction

It is typical for behavior of Asiatic black bear of the Russian Far East* (Ursus thibetanus)* to eat the cambium of coniferous trees, especially* Abies nephrolepis* [1–3]. A similar phenomenon was noted for black bears* (U. americanus)* in North America [4–8], brown bears* (U. arctos)* in North America [9], Europa [10–12], and Asia [13], and Japanese black bears* U. thibetanus japonicus* [14, 15]. In Japan animals hurt 17 species of coniferous trees and they are considered harmful to the country's forest economy [14].

The reasons for this phenomenon are studied insufficiently. Bazylnikov suggested that the cambium is a new kind of feed for Asiatic black bear, which it began to consume because of decrease of the volume of the main type of food—nuts and Korean pine cedar* (Pinus koraiensis)* [2]. However, this suggestion does not seem believable, since the total weight and caloric of eaten cambium are small, despite the fact that the bears spend a lot of time and energy to get it. A common opinion is that bears consume the cambium because of its high content of simple carbohydrates, such as glucose, fructose, and sucrose, especially in spring and summer, which makes this kind of feed attractive because of the high energy [9, 15–17]. It was shown that bears are attracted by terpenes [18], in particular, alpha-pinene (Yoshimura and Fujioka 2010). It remains an open question what is the additional reason of cambium feeding except that to get carbohydrates. Cambium of tree species Hinoki cypress* (Chamaecyparis obtusa)* and Japanese cedar* (Cryptomeria japonica)* is used by bears for feeding. Japanese researchers found in cambium the following compounds: *α*-Cubebene, *α*-Copanene, *α*-Longipinene, Isolongifolene, *α*-Cadinene, and *α*-Muurolene. Importance of these compounds for the bears has not yet been revealed [19].

The main goal of this study is to understand the phenomenon of selective eating of cambium by bears. The objectives of the work are to describe food-procuring behavior of bear aiming at the consumption of cambium; study damage of trees after consumption of cambium by bears; identify preferences of bears for cambium of different species of tree in different regions; and consider the possible causes of the consumption of this type of feed.

## 2. Methods

### 2.1. Study Area

**Figure 1:**
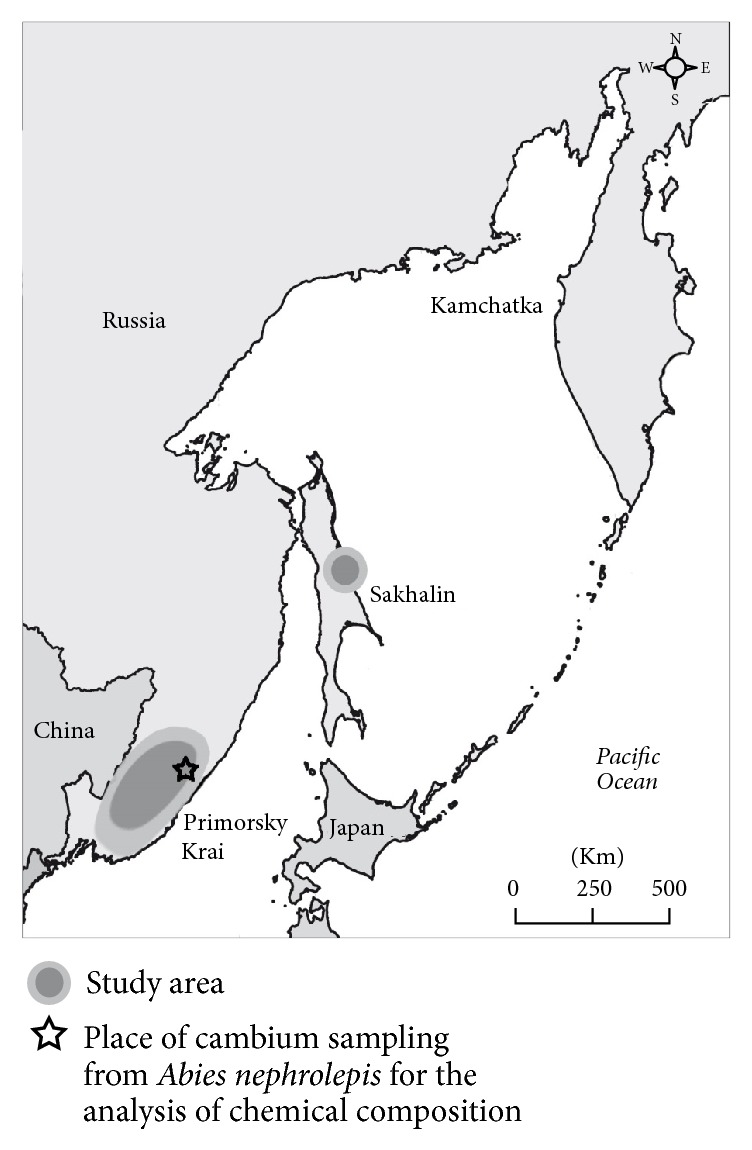
Map of observations was made during the period from 1998 to 2015.

The studies were conducted at locations in Primorsky Krai (Terneisky, Pozharsky, Krasnoarmeysky, Chuguevsky, Yakovlevsky, Nadezhdinsky, Ussuriysky, and Khasansky district) and the Sakhalin Island (Smirnykh district) (Figure 1). Observations were made during the period from 1998 to 2015.

The predominant natural land cover is coniferous forest in Primorsky Krai composed of Korean pine* (Pinus koraiensis)*, Manchurian fir* (A. nephrolepis)*, Dahurian larch* (Larix dahurica)*, and Ajan spruce* (Picea ajanensis)*. Deciduous and mixed forests also occur in this region, primarily at lower elevations, and include trembling aspen* (Populus tremula)*, Maximovich's poplar* (P. maximowiczii)*, Mongolian oak* (Quercus mongolica)*, Asian white birch* (Betula platyphylla)*, Manchurian alder* (Alnus hirsuta)*, and painted maple* (Acer mono)*. On the Sakhalin Island, the area of research was in Vengery the river basin. Mountain forests presented there with the maximum height of 1,500 m above sea level and include both coniferous and deciduous tree species, such as Sakhalin fir* (Abies sachalinensis)*, Ajan spruce, Siberian dwarf pine* (Pinus pumila)*, Erman's birch* (Betula ermanii)*, trembling aspen, and Manchurian alder.

Two species of bears (brown bear and Asiatic black bear) inhabit Primorsky Krai. The basis of feeding of both species in this region during the summer is an herbaceous vegetation and in the fall the nuts of the Korean pine and acorns of the Mongolian oak. Only brown bears inhabit Sakhalin Island. Their diet in addition to grassy vegetation includes the Pacific salmon [20].

### 2.2. Data Collection

We used methods of description for tracing bear activity, visual observation of bark stripping, and notification cases of cambium consumption by bears. For the tree that was identified as used for eaten, the species of the tree was recorded along with the diameter at breast height (dbh) using a tape. Features of the marks were recorded, including height, the circumference of damage, and data of the cambium composition. Also we count amount of the trees that become dry after the bears destroy the bark of the tree. Mass-spectrometric analysis of the cambium sample that was eaten by bears was done.

### 2.3. Analysis of Cambium Chemical Composition

For mass-spectrometric analysis we collected cambium of tree species* A. nephrolepis* most eaten by bears in Terneisky district of Primorsky Krai in April 2013 (Figure 1). Cambium was extracted with the 70% EtOH in the duck place at the temperature 4°C during 24 hours. Extracts after filtration were used for the mass-spectrometry analysis. ESIMS spectra were recorded with a 6510 LC Q-TOF (Agilent, USA) mass spectrometer with a dual electrospray-ionization source. All spectra were acquired in the negative- and positive-ion mode, with precalibration with a standard “HP-mix” for positive-ion mode. The capillary voltage was set to 3500 V, and the drying gas temperature was 325°C. The fragmentor voltage was set to 200 V.

## 3. Results

Studies have shown that bears consume the cambium only in a certain period of the year: from second half of May to August inclusive. But during this period the activity of the animals in relation to this type of food is not the same for different species of trees. So with respect to the cambium of fir* A. nephrolepis* the peak intensity in Primorsky Krai for Asiatic black bears is in June and the first half of July. On Sakhalin, we observed intensive feeding cambium hardwood trees throughout July and early August.

Bear gets access to cambium by stripping the bark from the tree. For this, bears bite it, sometimes stand up on his hind legs, and then pull from the top to down (sometimes vice versa) removing scraps. The animal licks the flowing sap and from the bare surface peels the cambium layer by cutters leaving on the trunk longitudinal grooves 10–30 cm long and 1–4 mm deep. Traces of several teeth are located together.

**Figure 2:**
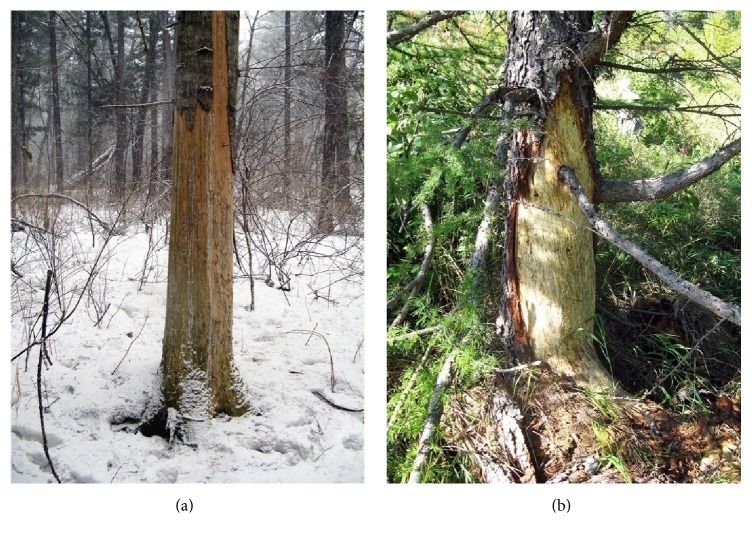
Coniferous trees, bark stripped by bears to get the cambium in Primorsky Krai: (a)* Abies nephrolepis* and (b)* Larix dahurica*.

**Figure 3:**
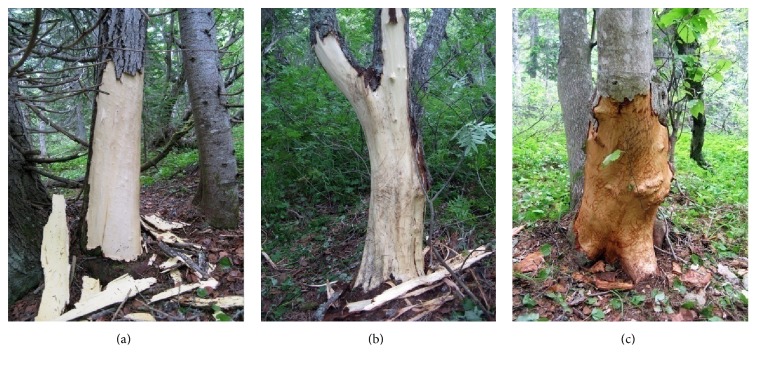
Deciduous trees, bark stripped by bears to get the cambium in Sakhalin Island: (a)* Populus tremula*, (b)* Salix caprea*, and (c)* Alnus hirsute*.

Trees that were damaged by bears have characteristic symptoms and were easy to recognize for many years (Figures 2 and 3) [3]. We made measurements of exempt parts of the cortex at 231 fir* A. nephrolepis*. Average damage for tree is 55.6% of the circumference of the base of the tree with a minimum of 13.3% and a maximum 100% cutters of circumference. According to our data twice damage was observed on the same tree 27 times. In 15 cases, these injuries were done at the same time and in 12 cases twice on the same trees but at different times. Bears were the reason of death of 31.4% of the surveyed trees that were attacked by them. All the trees died in the case when the bark was torn off round, and 15 trees had damage from 70.5 to 99.3% of the tree base circle. Thirteen trees were ragged on 77.4–87.9% of the circumference and survived after such damage, but some of them looked like suppressed. Trees that have damage up to 70% apparently looked healthy. Areas of trees, deprived bark (*n* = 258), had the following average dimensions of damage: height: 112.2 cm (from 30 to 231 cm), width: 52.7 cm (from 12 to 136 cm), and square: 0.7 (from 0.04 to 2.2 m^2^). The average diameter of the damaged trees was 28.4 cm (from 16 to 42 cm). It is noteworthy that the bears do not use trees with a diameter less than 15 cm and clearly prefer old-growth plants.

In the south of the Far East, eating the cambium is typical mostly for Asiatic black bear. The vast majority of damaged trees are the result of activities of this species. Nevertheless, eating the cambium by brown bear was also observed in some of Primorsky Krai [13]. On Sakhalin all the characteristic damage of the trees belongs to the brown bear.

Mostly bears used fir* A. nephrolepis* cambium (Figure 2(a)) in the Primorsky Kray. Among other damaged species of conifer trees we noted* Pinus koraiensis*,* Picea ajanensis*, and* Larix dahurica* (Figure 2(b).). In the south of Primorsky Krai is often damaged* Abies holophylla*. Among the hardwoods mentioned bears eat the cambium of trembling aspen in Primorsky Krai. Preference of fir trees over other types is perhaps due to the fact that the separation of the bark from them is relatively easy.

On Sakhalin Island, we observed a brown bear eating the cambium of four tree species: Sakhalin fir* Abies sachalinensis*,* Populus tremula*,* Salix caprea*, and* Alnus hirsuta* (Figure 3).

Analysis of cases of damage to trees with a view to obtain their cambium tissues shows that geographical distribution is inhomogeneous in different years. Our research also showed that in some areas there is increased consumption of cambium by bears in some years, whereas in the neighboring areas with the same population density of bears, this phenomenon cannot be observed. The following year, the situation changes frequently, and consumption of trees cambium by bears may not occur or move to another region.

Analysis of the chemical composition of the cambium* A. nephrolepis* revealed large amount of compounds that possesses tonic effect. The main ones are caffeoylquinic acid and its derivatives and quinic acid and its derivatives (Table 1). Also chemical composition of the cambium includes also large amounts of different antioxidants.

## 4. Discussions

It is interesting that bears consume the cambium tree sporadically: unequally in the area and to a varying extent in different years. Abramov et al. [1] indicate that, in some areas of Primorsky Krai, this phenomenon is not found. In Japan, the trees are damaged by bears in some areas and only in the artificial plantations [14]. In North America the black bear eating the cambium is not everywhere [5]. The reasons for such irregularities are apparently occurring in the bears that need certain important substances for life in conditions of shortage in the available feed. While the bears feed on cambium usually, they massively consume only one type of food (herbaceous plant in Primorsky Krai and salmon on Sakhalin Island) and it can be cause of lack of certain nutritional substances and induce feeding of cambium to get this compound.

Chemical analysis revealed several groups of compounds in the cambium extract. Caffeic, quinic, and ferulic acid and their derivatives are strong antioxidants and also possess a pronounced antifungal effect [21]. These compounds are powerful antioxidants and moreover may interact with opioid receptors, which are actively involved during the presser hibernation [22–24], and chlorogenic acid can ameliorate mechanical and cold hyperalgesia partly by activating GABAergic transmission in the spinal cord [25]. Quercetin is strong antioxidant, it also inhibits the activities of protein kinase enzyme, and it activates both estrogen receptor alpha and beta. Apigenin has a great number of biological activities; it not only possesses anti-inflammatory, anticancer, antioxidant, and antiallergic properties, but also inhibits enterovirus replication [26]. Compound epicatechin-3-gallate is ordinary for the green tea. It possesses antiradical properties and has a big number of biological activities. Glucaric acid supports the detoxification process of glucuronidation. Moreover cambium consists of oligo- and polysaccharides such as galactooligosaccharides, cellulose, lignin, and callose.

The prevailing opinion is that the bears eat the cambium to get energy from the sugar contained therein. Nevertheless, we believe that it is not the only and perhaps not the main reason. According to the results of the chemical composition analysis of the cambium we suggest that the main purpose of the use of this kind of food is tonic effect, recovery, and maintaining of the normal intestinal microbiota during the change of diet during the offseason. In support of this theory are the recent studies. It was described that during hibernation intestinal bacterial diversity of bears greatly reduced and moreover the species composition of the microbiota changes significantly during changing of feeding [27–29]. This means that between different feeding periods the bears need to restore their microbiota and they need efficient digestion. The presence of galactooligosaccharides, cellulose, lignin, and callose in the cambium is very favorable for growth of bacterial phylum Firmicutes that predominates in summer time.

Thus the substances contained in the cambium are very important to bear to maintain the normal operation of the intestinal microbiota and its recovery after the possible physiological disruptions. Although the source of these compounds could be other food, only the cambium has not excess medium for pathogenic bacteria, and the content of required compounds is so large. This explains the fact that despite the complexity of getting cambium, the bears spend much energy to get it.

## Figures and Tables

**Table 1:** The results of chemical analysis of the cambium from *Abies nephrolepis*.

Peak	*λ*max (nm)	Molecular ion	MS^2^ *(m/z)*	MS^3^ *(m/z)*	Tentative identification
1		191 [M-H]^−^	[191]: 173 (74.9), 127 (100), 111 (47.0), 109 (42.9), 85 (70.2)	163 (30.8), 125 (14.6), 109 (69.7), 99 (100)	Quinic acid
2	226, 294, 318	377 [M + Cl^−^]^−^, 683 [2M-H]^−^, 719 [2M + Cl]^−^, 707 [2M + Na]^+^	[341]: 215, 179, 113, 101		Caffeic acid hexoside
3	230, 266	533 [M-H]^−^	[533]: 191 (100)		Quinic acid derivative
4	322	309 [M-H]^−^	[309]: 193 (100), 291 (45.3), 133 (10.3)		Feruloyl-L-malic acid
5	336, 276	293 [M-H]^−^			Apigenin
6		133 [M-H]^−^	[133]: 115 (100)		Malic acid
7	365	383 [M-H]^−^			Rosmarinic acid
8	241, 300, 324	353 [M-H]^−^	[353]: 191 (100)	[191]: 173 (22.7), 171 (21.9), 127 (100), 111 (52.4), 109 (28.1), 85 (47.8)	3-O-Caffeoylquinic acid
9	242, 300, 325	353 [M-H]^−^	[191]: 173 (55.2), 127 (100), 109 (65.8), 85 (97.0)		5-O-Caffeoylquinic acid-hexoside
10	324	353 [M-H]^−^	[353]: 191 (100)		Chlorogenic acid
11		325 [M-H]^−^	[325]: 265 (12.2), 187 (43.1), 163 (100), 145 (95.9), 119 (19.2)		*p*-Coumaric acid-O-hexoside
12	355	301 [M-H]^−^	[301]: 273.0399 (13), 229.0504 (3), 178.9983 (48), 151.0029 (100), 121.0292 (15)		Quercetin II
13	326	397 [M-H]^−^	[397]: 134 (25.3), 175 (21.9), 193 (100), 217 (44.4), 337 (32.6)		Ferulic acid derivative
14	304		[353]: 191 (100)	[191]: 179 (100), 127 (44.8), 111 (47.2), 93 (85.3), 85 (85.7), 81 (26.4)	Cis-5-O-*p*-coumaroylquinic acid
15			[329]: 311 (25.7), 293 (21), 229 (100), 211 (81.4), 171 (40)		Trihydroxyoctadecenoic acid
16		297 [M-H]^−^			Galacturonic acid 1-phosphate
17	328, 298	311 [M-H]^−^	[311]: 133.0 (100), 115.0 (37)		Caftaric acid
18		327 [M-H]^−^	[327]: 291 (56.0), 229 (100), 211 (51.9), 209 (10.8), 171 (62.1), 165 (15.2)		Oxo-dihydroxy-octadecenoic acid
19	240	441 [M-H]^−^	[441] 289, 245, 169, 125		(−)-Epicatechin-3-gallate
20	223	934 [M-H]^−^	[934]: 915 (53.9), 897 (77.7), 783 (46.9), 633 (71.1), 301 (100)		Galloyl-bis-HHDP-O-hexoside
21		455 [M-H]^−^	[455]: 306 (100), 288 (34.8), 272 (11.6), 160 (16.4)	[306 → 254]: 210 (40.6), 179 (100), 161 (43.7), 135	Caffeic acid derivative
22	324	193 [M-H]^−^	[193]: 178 (17.8), 149 (100)		Ferulic acid
23		485 [M-H]^−^			Kaempferol-3-glucuronide
24		425 [M-H]^−^	[425]: 327 (100), 209 (21.9)		Glucaric acid derivative
25	245	175 [M-H]^−^	[175]: 115 (100)		L-Ascorbic acid
26		207 [M-H]^−^			Phosphocholine
27	327, 300, 268	295 [M-H]^−^	[295]: 277 (10.0), 179 (76.5), 133 (100), 115 (21.9)		Caffeoylmalic acid
28	334, 365	209 [M-H]^−^	[209]: 191 (100), 85 (25.9)		Glucaric acid
